# Winning the Lottery: A Simulation Study Comparing Scarce Resource Allocation Protocols in Crisis Scenarios

**DOI:** 10.7759/cureus.76977

**Published:** 2025-01-05

**Authors:** Joshua S Sohmer, Sabina Fridman, Darian Peters, Mario Jacomino, George Luck

**Affiliations:** 1 Medicine, Florida Atlantic University Charles E. Schmidt College of Medicine, Boca Raton, USA; 2 Pediatrics, Memorial Healthcare, Pembroke Pines, USA; 3 Women's and Children's Health, Florida Atlantic University Charles E. Schmidt College of Medicine, Boca Raton, USA; 4 Integrated Medical Science, Florida Atlantic University Charles E. Schmidt College of Medicine, Boca Raton, USA

**Keywords:** crisis allocation, healthcare ethics, pandemic response, simulation study, triage algorithms

## Abstract

Introduction

In times of crisis, such as natural disasters, pandemics, or other emergencies, healthcare facilities often experience an unprecedented surge in critically ill or severely injured patients. When the demand for life-saving resources surpasses the available supply, healthcare leaders must implement scarce resource allocation (SRA) protocols, which are defined by state governments or hospital committees. Due to the lack of federal standardization and the wide variations in these protocols across states and healthcare systems, researchers aimed to investigate the disparities in SRA protocols and their impact on patient outcomes in preparation for future emergencies. ­­

Methods

Researchers created a simulation involving mock patients admitted to a hospital with limited ventilator availability, where they were required to implement an SRA protocol. Nine mock adult patient profiles were generated, each varying in age, biological sex, past medical history, social history, and illness acuity and severity. Researchers also comprehensively reviewed SRA protocols implemented across the United States during the COVID-19 pandemic. Six protocols were selected and applied to the mock patient population. Variations in the methodology of allocation and outcomes of resource stewardship were observed.

Results

Significant differences were found among the six SRA protocols, including differences in objective scoring categories, exclusion criteria, considerations for age and pregnancy, tie-breaking methods, and the use of lottery systems. These protocol differences influenced the outcomes of life-saving treatments received by different patients. In this simulation, no two state algorithms provided the same ventilator allocation results for the nine patients.

Conclusion

SRA protocols either emphasized scoring systems or employed an ambiguous lottery system, placing an unnecessary burden on physicians and patients. As a result, the researchers advocate for federal standardization of SRA protocols, to ensure equal access to critical medical care for all individuals, regardless of location, and to eliminate the element of chance that currently varies by state.

## Introduction

Throughout history, crises such as wars, natural disasters, pandemics, epidemics, and other emergencies have had profound impacts on healthcare systems and societies. These events leave lasting effects, shaping our responses to infectious diseases, conflicts, and emergencies [1]. Understanding past crises is essential for grasping the complexities of resource allocation during healthcare emergencies [2].

Previous pandemics, including the Spanish flu and the HIV/AIDS crisis, have revealed recurring challenges and evolving strategies for managing healthcare demands [3]. Historical context provides valuable insights into resource allocation during healthcare crises. When an outbreak, epidemic, or pandemic is declared, uncertainties arise, especially when novel infectious agents are involved. These uncertainties include infection rates, symptom severity, treatment needs, and mortality rates [4].

Beyond pandemics, other crises such as wars and natural disasters pose unique challenges for healthcare systems. For instance, wars lead to an influx of critically ill individuals, putting significant strain on healthcare infrastructure [5]. Natural disasters like earthquakes and hurricanes further disrupt healthcare facilities, creating a sudden surge in demand for medical resources [6].

The World Health Organization (WHO) plays a vital role in monitoring global disease activity and operates through a network of international centers [7]. While primarily focused on infectious diseases, the WHO also aids healthcare systems in conflict zones, addressing public health during humanitarian crises [8]. These agencies analyze data to identify factors and uncertainties, enabling healthcare organizations to prepare for surges in life-saving resource demands that often exceed normal requirements [9].

In the United States, the early months of the COVID-19 pandemic demonstrated the challenges of handling unprecedented patient surges. Hospitals across the country faced critical resource shortages in a short time frame [10]. With unclear guidelines, healthcare systems struggled to respond, highlighting the urgent need for robust preparedness and pandemic response strategies [11].

During resource shortages, healthcare systems implement crisis standards of care, also known as scarce resource allocation (SRA) protocols [12]. These protocols provide an ethical framework for decision-making in crises, but they lack standardization and vary significantly across states and healthcare systems [13]. A 2020 study highlighted the lack of uniformity among US SRA protocols, with only a few states aligning with ethical guidelines set by the Institute of Medicine (IOM) and the CHEST Task Force Executive Committee [14].

To address these concerns, our study uses a simulation to examine the impact of disparate SRA protocols on patient outcomes during resource-scarce scenarios. By simulating medical resource scarcity in epidemic or pandemic conditions, this research aims to reveal how treatment and outcomes vary under different SRA frameworks. Our study contributes to understanding resource allocation in healthcare crises and informs efforts to enhance global preparedness and response strategies.

## Materials and methods

Participants

Nine mock adult patient profiles were created to represent critically ill patients requiring ventilators. The profiles, with varying ages (27-92 years), biological sex, past medical history (PMHx), and social history (SHx), were randomly generated by a blinded researcher to reflect the diversity of patients healthcare providers might encounter during a crisis (Table 1). This diversity aims to replicate the real-world complexities of resource allocation decisions in scenarios such as infectious disease outbreaks, natural disasters, or wartime scenarios.

**Table 1:** Mock COVID-19-positive patient demographics and SOFA scoring PMHx: previous medical history, SOFA: sequential organ failure assessment, SES: socioeconomic status, SHx: social history.

Patient #	Age	Sex	SOFA Score	PMHx	SHx (if applicable)
1	45	F	14	No significant PMHx	Nurse
2	65	M	10	End-stage renal disease	O
3	27	F	10	30 weeks pregnant	O
4	50	M	6	Hypertension controlled on medication	Low SES
5	92	M	4	Dementia	O
6	40	M	8	Metastatic breast cancer	O
7	28	F	5	No significant PMHx	Firefighter
8	57	M	2	Late-stage multiple sclerosis	O
9	75	F	8	Heart failure patient class IV	O

Blinded procedure

To maintain objectivity, the researcher responsible for creating the mock patient profiles did not participate in the application of SRA protocols. This blinding procedure minimized potential bias, ensuring that the profiles were developed independently of the protocol applications.

Materials

Six SRA protocols implemented during the early stages of the COVID-19 pandemic in the United States (March 2020) were included in the study. These protocols were selected from diverse regions and institutions to capture varying approaches to resource allocation: the American College of Chest Physicians (MD) [12], University of Pittsburgh Medical Center (PA) [13], Baptist Health System (FL) [14], New York State Department of Health (NY) [15], Health Care Crisis Nebraska Medical Emergency Operations Center (NE) [16], and California Department of Health (CA) [17]. These protocols represent both government- and hospital-specific guidelines used to address ventilator shortages during the pandemic.

Procedure

The simulation was conducted in a hospital setting with 30 ventilators, of which only three remained available for nine patients needing ventilation. The six SRA protocols were applied to prioritize ventilator allocation among these patients.

Each protocol employed different methods for prioritization: some assigned a quantitative rank, others qualitative priority groups (e.g., high, medium, and low), and some excluded patients based on specific criteria. This variability in how patients were prioritized reflects real-world differences in SRA protocols and allows for a comprehensive analysis of allocation approaches.

All simulated patients were insured US citizens or legal residents, with insurance status and legal status excluded as factors in triage. Two investigators independently applied the six protocols to the nine patients to minimize bias and ensure consistency. Any discrepancies between investigators were resolved through discussion and consensus.

Scoring description

All six protocols utilized the Sequential Organ Failure Assessment (SOFA) scoring system as part of their prioritization criteria. The SOFA score is an objective tool that evaluates the severity of organ failure based on clinical data, with scores ranging from 0 to 24. However, the application of the SOFA score varied among the protocols. Some protocols set specific thresholds where higher SOFA scores indicated a poor prognosis, prompting prioritization of patients with lower scores. Other protocols combined SOFA scores with additional factors, such as age, comorbidities, or the likelihood of recovery, to guide decisions on patient prioritization for ventilator access.

Exclusion criteria

Exclusion criteria, which deprioritize patients with severe comorbidities or poor prognoses, were included in some SRA protocols. Specific comorbidities such as advanced heart failure or incurable metastatic cancer were listed as exclusions in several protocols. However, the exact exclusions varied across the protocols, reflecting differences in how patient survivability and potential for recovery were assessed.

Qualitative review and criteria for analysis

A qualitative review of each protocol's inclusion and exclusion criteria was conducted to identify key variations and evaluate their impact on patient outcomes. Several factors were considered for this analysis, including exclusion criteria, age, pregnancy, tie-breaking mechanisms, lottery systems, and considerations for healthcare workers and first responders.

The SOFA score, a crucial element in most SRA protocols, was calculated for each mock patient based on vital signs and laboratory values provided in the patient profiles (Table 1). The SOFA score was originally developed to predict outcomes in septic patients. SOFA scores provided an objective measure to assess and prioritize patients across protocols.

## Results

Variation among protocol algorithms

A qualitative review of the six selected protocols revealed significant differences in how patient priority was assigned during resource allocation crises. These differences are summarized in Table 2 and include variations in scoring systems, exclusion criteria, and considerations for factors such as age, pregnancy, and occupation.

**Table 2:** Variation among protocol algorithms MD: College of Chest Physicians in Maryland, PA: University of Pittsburgh Medical Center, FL: Baptist Health System in Florida, NY: New York State Department of Health, CA: California Department of Health, NE: Health Care Crisis Nebraska Medical Emergency Operations Center.

Components of patient priority assignment	MD	PA	FL	NY	CA	NE
SOFA score	✓	✓	✓	✓	✓	✓
Exclusion criteria	✓	O	✓	✓	O	O
Age consideration	✓	Only in ties	O	O	✓	O
Pregnancy consideration	✓	O	O	O	✓	O
Healthcare worker/first responder consideration	O	Only in ties	O	O	✓	O
Tie break/lottery system	✓	✓	✓	✓	✓	O

While all protocols utilized the SOFA score to prioritize patients, their application varied. For example, some protocols set higher SOFA score thresholds to classify patients as having a poor prognosis, such as MD (SOFA > 14), while others used stricter cutoffs, like NY (SOFA > 11). These variations highlight how the same scoring system can lead to different prioritization strategies.

Exclusion criteria were implemented in MD, FL, and NY protocols to deprioritize patients with severe comorbidities or poor prognoses. Specific conditions, such as advanced heart failure or incurable metastatic cancer, were consistently cited, but the details differed among these protocols. By contrast, exclusion criteria were absent in the PA, CA, and NE protocols.

Age and pregnancy considerations also varied significantly. Younger patients were explicitly prioritized in the MD and CA protocols, while the PA protocol considered age only as a tiebreaker. The FL, NY, and NE protocols did not account for age. Pregnancy was prioritized in the MD and CA protocols by subtracting points from pregnant patients’ overall priority scores, but the remaining protocols did not consider pregnancy in allocation decisions.

Healthcare worker or first responder status was another point of divergence. Only the CA protocol temporarily exempted frontline workers, such as police officers, firefighters, and healthcare workers, from allocation scoring, while the PA protocol considered occupation only as a tiebreaker. The MD, FL, NY, and NE protocols did not factor occupation into prioritization.

Tie-breaking mechanisms further demonstrated variability. MD, FL, and NY protocols employed a first-come, first-served approach when patients had equal priority scores. By contrast, PA and CA protocols recommended a randomized lottery as a final method of resolution. The NE protocol did not specify any tie-breaking or lottery mechanism, leading to potential ambiguity in patient prioritization.

Outcomes of patient ventilator allocation by SRA protocol

In this simulated resource-limited scenario, only one patient consistently received a ventilator allocation in five of the six protocols (patient #7), a 28-year-old first responder with no significant medical history (Table 3). Although it is worth considering that had NE provided more detailed information on prioritization, this patient would also have been a candidate for the highest priority in their algorithm.

**Table 3:** Patient ventilator allocation by SRA protocol *** = allocated ventilator, ** = tied for ventilator but lost due to tie-break measures, * = qualified for ventilator but protocol lacks tie-breaking instructions to determine allocation, O = did not qualify for a ventilator. MD: College of Chest Physicians in Maryland, PA: University of Pittsburgh Medical Center, FL: Baptist Health System in Florida, NY: New York State Department of Health, CA: California Department of Health, NE: Health Care Crisis Nebraska Medical Emergency Operations Center.

Patient	Age	Sex	SOFA	MD	PA	FL	NY	CA	NE
1	45	F	14	O	O	O	O	***	O
2	65	M	10	O	O	O	O	O	O
3	27	F	10	***	O	O	O	***	O
4	50	M	6	O	***	***	**	O	*
5	92	M	4	**	***	***	***	O	*
6	40	M	8	***	O	O	O	O	*
7	28	F	5	***	***	***	***	***	*
8	57	M	2	O	O	O	***	O	*

The primary determinants for definitive ventilator allocation were a lower SOFA score and no significant comorbidities. These concepts are exemplified by patient #5 (a 92-year-old male patient with dementia, who had a SOFA score of 4) and patient #7 (a 28-year-old female patient with no significant history, who had a SOFA score of 5). Both of these patients consistently received the most ventilators across all six protocols.

However, patients with higher SOFA scores, signs of organ failure, or metastatic cancer were more likely to meet exclusion criteria or be deemed lower priority for ventilator allocation. For example, patient #1, who had the highest SOFA score among all patients, consistently received a low priority for ventilation allocation except in CA, where she was prioritized solely due to her occupation as a nurse. However, she was ranked for priority in two of the six algorithms due to her high SOFA score of 14.

Outcomes vary by state due to differences in listed exclusion criteria within each protocol, rather than solely based on medical necessity. For example, patient #8, a 57-year-old male patient with advanced multiple sclerosis with a SOFA score of 2, was immediately excluded from the scarce resource allocation lottery under Baptist’s protocol due to his medical history; however, he qualified for a ventilator under the NY protocol due to his SOFA score.

## Discussion

The allocation of critical healthcare resources, particularly in scenarios of scarcity, is a complex and ethically charged issue that demands careful consideration and reflection [18]. Variability in SRA protocols raises concerns about the fairness and consistency of resource distribution [11]. This simulation-based study reveals that such variability can introduce an element of chance, resembling a lottery, despite efforts to minimize uncertainty [12].

When demand exceeds supply, decisions regarding ventilator allocation become life-or-death matters. These decisions should not be influenced by location, timing, or luck [19]. However, this study demonstrates that variability in SRA protocols can result in situations where receiving the last ventilator depends more on the “luck” associated with a hospital’s location. Geographical disparities have led to significant chances of receiving life-saving treatments, depending solely on the state in which care is provided in the United States [19].

Several factors contribute to geographical disparities, further highlighting inconsistencies in the standards of care. Variables such as pregnancy, healthcare worker status, long-term survival assessment, pre-hospital functional status, and exclusion criteria produced varying outcomes for our mock patients depending on their geographical location. The primary criteria for allocating ventilators, the SOFA score, which was originally designed for sepsis prediction, may not accurately capture the medically complex needs of patients within resource-limited scenarios [20].

The utilization of a lottery system for resource allocation and the associated exclusion criteria introduce inherent ethical issues [19]. For instance, some mock elderly patients had better outcomes in New York, where the protocol aims to prevent age-based discrimination and promote allocation based on the estimated likelihood of survival. However, in some states, hospital arrival time played a significant role as a tie-breaker for ventilator allocation decisions. This measure raises concerns over the potential negative impact on individuals, particularly those of lower socioeconomic status (SES), who may face challenges in timely arrival. For example, individuals relying on public transportation rather than opting for private transportation or ambulance services may encounter external factors jeopardizing their chances of being allocated a ventilator.

The first-come, first-served approach, rather than being a medical prioritization, may further exacerbate disparities among those of low SES who may avoid medical attention due to a generational distrust of the healthcare system [21]. Additionally, individuals of low SES may not have medical insurance, leading to delayed seeking of medical care to avoid substantial bills [21]. Access to life-saving treatment should not depend on the specific hospital policy one happens to encounter. This variability should never apply to life-saving treatment. Standardized and equitable protocols are essential to ensure that all individuals, regardless of location or specific hospital policy, access critical medical resources equally.

A common pattern observed between the protocols was that patients with fewer, less life-threatening comorbidities were more often allocated ventilators than patients with more significant past medical histories. This raises considerable ethical questions: Are the lives of those with multiple sclerosis or dementia not worth saving over those with minimal comorbidities? Should a middle-aged woman with incurable metastatic breast cancer and a life expectancy of 1-2 years be excluded? These are challenging and sensitive considerations that underscore the need for clear and equitable guidelines in resource allocation.

Another crucial consideration is allocating resources to medical workers or first responders, as observed in states like California. Patients who might not receive a ventilator in other states due to a poorer prognosis were guaranteed treatment due to their occupation. This raises ethical questions regarding the role of employment in determining care and whether it is ethical to prioritize individuals based on their occupation [22].

Finally, when examining the allocation of resources to a pregnant patient, it is evident that there is a lack of standardized guidance [23]. We observe that pregnant patients only received a ventilator in two states, highlighting the need for clear and standardized guidelines that consider the unique circumstances surrounding these patients. Authors believe that it would be unlikely for any physician to withhold access to life-saving treatment to a pregnant patient under any circumstance. There are many reasons that standardization is essential in healthcare resource allocation, and the scenario of the pregnant patient highlights that inadequacies may exist across state guidelines. This leaves physicians to decide on the validity of prioritizing one life over two without clear and sufficient guidance [23].

Limitations

It must be addressed that this study possesses several limitations that should be considered when interpreting its findings. Utilizing mock patients focusing on a specific resource may not fully reflect the real-life dynamics and complexities of resource allocation during varying crisis scenarios, whether stemming from infectious disease outbreaks, natural disasters, or wartime scenarios. Additionally, this study’s generalizability was limited by a relatively small sample size of simulated patients, only analyzing six SRA protocols, and an arbitrary ventilator count. While a sample size of nine mock patients was suitable for a simulation, a larger sample size could enhance the strength of the study’s findings. Increasing the number of protocols would provide a more comprehensive view of resource allocation across the United States in different crisis settings.

It should also be considered that the protocols utilized for this study were those active in 2020 during the COVID-19 pandemic, while protocols may have been updated since then. Therefore, these findings may not fully represent the current resource allocation state across various crisis scenarios. Furthermore, this scenario’s use of an arbitrary count of available ventilators (3 of 30) does not reflect potential variations in the availability of resources across different healthcare facilities, a significant variable in allocation decision-making. Additionally, the study did not consider the type of ventilators owned by hospitals. There are full-featured mechanical ventilators, and approximately half of these are pediatric/neonatal capable (for individuals under 14 years old). As of 2010, there are an estimated 98,738 devices other than full-feature ventilators at US acute care hospitals, which adds yet another layer of complexity to resource allocation [24].

Another critical aspect to consider is the exclusion of factors that may influence the effectiveness of imposed SRA protocols, such as institutional capacity and public perception of fairness. Additionally, hospitals with limited staffing or proper infrastructure may face barriers to implementing protocols consistently, or public perceptions of bias or inequity in resource distribution could undermine trust or compliance during times of crisis. 

Finally, our simulated scenario did not include pediatric patients or other social and economic factors, including but not limited to medical insurance, socioeconomic disparities, or legal status in the country, which may complicate further decision-making. In the case of a pediatric patient, it would be pertinent to seek the involvement of an ethics committee, a dimension not explored in this simulation study but crucial to ensure legal and ethical compliance in healthcare resource allocation.

Overall, while this study provides valuable insight into variations among SRA protocols in the allocation of resources, these limitations underscore the need for further research to expand the scope with the involvement of more protocols, larger sample sizes, and evolving healthcare guidelines to provide an increasingly comprehensive understanding of equitable resource allocation during crisis settings.

## Conclusions

This study highlights the extensive inconsistencies in SRA protocols and their outcomes, emphasizing the dire need for standardized national guidelines for equitable resource allocation. Variability in prioritization criteria, such as SOFA scores, exclusion criteria, and tie-breaking mechanisms, underscores the ethical and practical challenges faced by healthcare providers during crisis scenarios.

To address these issues, the development of a gold-standard SRA protocol is critical. Such a framework would minimize geographic and systemic disparities, reduce the burden on physicians, and ensure fair distribution of life-saving resources. While this simulation focused on ventilator allocation, the findings underscore the broader need for consistent, ethically sound protocols to guide resource allocation in all future crises. Preparedness and uniformity remain crucial to navigating the challenges of an unpredictable healthcare landscape.
